# The ADAM17-directed Inhibitory Antibody MEDI3622 Antagonizes Radiotherapy-induced VEGF Release and Sensitizes Non–Small Cell Lung Cancer for Radiotherapy

**DOI:** 10.1158/2767-9764.CRC-21-0067

**Published:** 2021-12-22

**Authors:** Fabienne Tschanz, Sabine Bender, Irma Telarovic, Verena Waller, Roberto F. Speck, Martin Pruschy

**Affiliations:** 1 Laboratory for Applied Radiobiology, Department of Radiation Oncology, University Hospital Zurich, University of Zurich, Zurich, Switzerland.; 2 Department of Infectious Diseases and Hospital Epidemiology, University Hospital Zurich, University of Zurich, Zurich, Switzerland.

## Abstract

The cellular response to ionizing radiation (IR) depends on tumor cell and microenvironmental factors. Here, we investigated the role of IR-induced ADAM17 matrix metalloproteinase activity for the intercellular communication between tumor cells and the tumor vasculature in non–small cell lung cancer (NSCLC) tumor models. Factors shed by ADAM17 from NSCLC tumor cells (A549, H358) and relevant for endothelial cell migration were investigated using transwell migration assays, ELISA, and flow cytometry. Tumor angiogenesis–related endpoints were analyzed with the chorio-allantoic membrane assay and in murine NSCLC tumor models. Efficacy-oriented experiments were performed in a murine orthotopic NSCLC tumor model using irradiation with an image-guided small-animal radiotherapy platform alone and in combination with the novel ADAM17-directed antibody MEDI3622. *In vitro*, VEGF was identified as the major factor responsible for IR-induced and ADAM17-dependent endothelial cell migration toward attracting tumor cells. IR strongly enhanced tumor cell–associated ADAM17 activity, released VEGF in an ADAM17-dependent manner, and thereby coordinated the communication between tumor and endothelial cells. *In vivo*, tumor growth and microvessel size and density were strongly decreased in response to the combined treatment modality of IR and MEDI3622 but not by either treatment modality alone and thus suggest that the supra-additive effect of the combined treatment modality is in part due to abrogation of the ADAM17-mediated IR-induced protective effect on the tumor vasculature. Furthermore, we demonstrate that the novel ADAM17-inhibitory antibody MEDI3622 potently improves the radiotherapy response of NSCLC.

**Significance:**

The tumor response to radiotherapy is influenced by several factors of the tumor microenvironment. We demonstrate that inhibition of the sheddase ADAM17 by the novel antibody MEDI3622 reduces IR-induced VEGF release from tumor cells relevant for endothelial cell migration and vasculature protection, thereby enhancing radiotherapy treatment outcome of NSCLC.

## Introduction

Cancer is a leading cause of death worldwide with lung cancer being the second most frequent type in men and women, respectively (1). Radiotherapy alone or in multimodality approaches is applied in approximately 50% of all patients with cancer with solid tumors (2, 3). Ionizing radiation (IR) induces the formation of reactive oxygen species that induce DNA damage and chromosomal aberrations which eventually result in mitotic catastrophe and cell death (4). Apart from cell damage, IR induces a complex network of secreted factors from irradiated cancer cells, stromal cells, and endothelial cells leading to a multilayered stress response. The storm of secreted factors can stimulate tumor outgrowth, dissemination, incomplete treatment responses, and immune reactions (5).

Furthermore, several studies demonstrated that IR promotes migration and invasion of the irradiated cells by influencing the microenvironment, cell–cell junctions, protease secretion, and the induction of epithelial-to-mesenchymal transition (6, 7). A significant part of this signaling is evoked by an IR-induced secretome, which is partly mediated by ectodomain shedding (8, 9). We recently identified the matrix metalloproteinase ADAM17 (a disintegrin and metalloproteinase 17) to be upregulated in response to IR in a time- and dose-dependent manner (10). ADAM17 is involved in the cleavage of extracellular domains of proteins from the cell surface and thereby releasing cytokines, growth factors, and numerous other substrates that influence cell growth, migration, and radioresistance (11). Substrates include TGFα, ectodomains of several receptor tyrosine kinases, ligands of the ErbB family, ALCAM, and many more (12). ADAM17 activity correlates with a poor prognosis and worse therapy outcome in NSCLC, which makes ADAM17 a promising target for cancer treatment in combination with irradiation.

Over the past decades, it has been established that the tumor vasculature not only promotes tumor growth but also influences treatment response (13). The combined treatment modality of radiotherapy with tumor vasculature–targeting agents is a well-established concept; however, treatment combinations of radiotherapy with antiangiogenic agents are barely clinically approved, often due to limiting normal tissue toxicities. Thus, novel strategies are of high demand, which might be targeting radiotherapy-induced and tumor-specific processes. The irradiation-induced signaling pathways evoked by ADAM17 as a mediator for intercellular communication and their implications for cancer growth and therapy still need to be elucidated. Although, a putative IR-induced and ADAM17-dependent release of factors regulating tumor vasculature formation and integrity makes ADAM17 an interesting target for a combined treatment modality with radiotherapy.

Here we investigate IR- and ADAM17-mediated intercellular communication between tumor and endothelial cells *in vitro*, and vessel formation with the angiogenesis-oriented chorio-allantoic membrane (CAM) assay *ex ovo*. We address ADAM17-inhibition with the novel ADAM17-directed inhibitory antibody MEDI3622 (14) in combination with IR in murine tumor models *in vivo*, including its antiangiogenic response, and probe the efficacy of the combined treatment of radiotherapy and MEDI3622 in an orthotopic lung adenocarcinoma tumor model. With these data, we demonstrate new insights on the interaction between the tumor and the tumor vasculature and on the relevance of ADAM17 as a target for a combined treatment modality with radiotherapy.

## Materials and Methods

### Cell Lines

Lung adenocarcinoma cells (A549: CCL-185, **RRID**: CVCL_0023 and NCI-H358: CRL-5807; **RRID**: CVCL_1559) were obtained from ATCC in, 2018. Cell line authentication (Eurofins) was performed by short tandem repeat profiling (Applied Biosystems AMPFLSTR Identifiler Plus) and compared using CLASTR 1.4.4. The lung adenocarcinoma cells were cultured in RPMI1640 media (Gibco; 22409–015). All media were supplemented with 10% (v/v) FBS (Gibco; 10270–106), 1% (v/v) penicillin–streptomycin (Gibco; 15140122) and 1% (v/v) l-glutamine (Gibco; 35050–38). The cells were cultured as monolayers in culture plates in a 37°C atmosphere containing 5% CO_2_ and passaged two times after thawing to perform experiments. Human umbilical vein endothelial cells (HUVEC: C-12203; **RRID**: CVCL_2959 obtained from Promocell, 2019) and human pulmonary microvascular endothelial cells (HPMEC: C- 12281 obtained from Promocell, 2019) were kept in endothelial cell growth medium (ECGM, Promocell; C-22215) and supplementary growth factors (Promocell; C-39210). All cell lines were tested every three to four months for *Mycoplasma* contamination using a commercial kit (MycoAlert, Lonza; LT07–118) and during microscopy with DAPI (Invitrogen; D1306, 1:25,000 in PBS) as DNA-intercalating dye.

### Stable Cell Lines

Custom lentiviral shRNA expression vectors were obtained from Cellecta. The expression vector used was pRSITPRP-U6Tet-sh-PGK-TetRep-2A-TagRFP-2A-Puro. Insert sequence for shADAM17_2 was ACC GGG ATC ATC GCT TCT ATA GAT ACG TTA ATA TTC ATA GCG TAT CTG TAG AAG CGA TGA TCT TTT, for shADAM17_3 was ACC GGC CTG GTT ACA ACT TAT GAA TTG TTA ATA TTC ATA GCA ATT CAT GAG TTG TAA CCA GGT TTT, and for the control cell line sh_NT was ACC GGC AAC AAG ATG GAG AGC ACT AAG TTA ATA TTC ATA GCT TGG TGC TCT TCA TCT TGT TGT TTT. The plasmid short hairpin RNA (shRNA) constructs were packaged into pseudotyped viral particles in HEK293FT cells (PTA-5077, RRID: CVCL_6911 obtained from ATCC, 2016), using the psPAX2/pMD2.G packaging plasmid mix (Cellecta; CPCP-K2A) according to the manufacturer's protocol. Target cells were transduced with lentiviral supernatants in presence of 5 μg/mL polybrene. Cells were selected in presence of 1 μg/mL puromycin. The induction of the directed shRNA construct always began 72 hours prior to starting the experiment with doxycycline (1 μg/mL, Sigma; D9891).

### Cell Treatment and Irradiation

Cells were pretreated with MEDI3622 or IgG1 (200 nmol/L, MedImmune, LLC) in PBS for 1 hour prior to sham treatment or irradiation with a RS-2000 225 kV irradiator at 4.2 Gy/minute (Rad Source). MEDI3622 concentration used was based on preliminary experiments with increasing concentrations ranging from 100 to 500 nmol/L. ADAM17 activity was efficiently downregulated with 200 nmol/L of MEDI3622. Where indicated, 50 ng/mL recombinant human VEGF-A (Sigma; V7259) and 250 μg/mL bevacizumab (Avastin, Roche) was added.

### ADAM17 Activity Assay

ADAM17 activity in cell lysates was determined using the InnoZyme TACE Activity Kit (Calbiochem; CBA042) according to the manufacturer's protocol. Cell were lysed using the CytoBuster Protein Extraction Reagent (EMD Millipore; 71009) followed by centrifugation at 15,000 rpm for 8 minutes. Protein concentration was quantified in the filtered supernatant using a NanoDrop spectrophotometer. Purified recombinant human TACE was used as a positive control. The lysates were incubated in an anti-human ADAM17 antibody precoated 96-well plate for 1 hour, followed by five washing steps. Detection solution containing an internally quenched fluorescent substrate was added for 4 hours and fluorescence was measured at an excitation wavelength of 324 nm and emission wavelength of 400 nm using a TECAN Infinite 200 PRO spectrophotometer. The level of fluorescence was directly related to the enzyme activity.

### qRT-PCR

Sample mRNA expression was determined 24 hours after irradiation. Cell lysate collection and RNA isolation were performed using a RNeasy Mini Kit (Qiagen; 74004) according to the manufacturer's instructions. RNA was reverse transcribed using High-Capacity cDNA Reverse Transcription Kit (Applied Biosciences; 4368814) and cDNA was amplified using SYBR Green Master Mix (Sigma; S9430) with the following primers: (5′-3′): GAPDH forward: AACGGATTTGGTCGTATTGGGC; GAPDH reverse: TTGATTTTGGAGGGATCTCG; VEGF forward: GAAGTGGTGAAGTTCATGGATGTCTAT; VEGF reverse: TCAGGGTACTCCTGGAAGATGTC. qRT-PCR was performed on LightCycler 480 (Roche).

### ELISA

Secreted VEGF-A concentration was collected 24 hours and 48 hours after IR and detected in filtered conditioned media using a VEGF-A DuoSet ELISA kit according to the manufacturer's guidelines (R&D Systems; DY293B-05). The absorbance was determined with a plate reader TECAN Infinite 200 PRO at 450 nm excitation and 540 nm emission and normalized to live cell count using EVE Automatic cell counter (NanoEnTek).

### *Ex Ovo* CAM Assay

Fertilized chicken eggs (Animalco) were cracked open in a plastic bowl (Thermoflex; 714.0) on developmental day 3 and incubated at 37°C at 60% humidity (Binder incubator; BIND_6019) and breathing air. On developmental day 9, 4 × 10^6^ tumor cells in 20 μL Matrigel (Corning; 354234) were explanted onto the CAM between major blood vessels. The shRNA-transfected tumor cells, which were doxycycline-induced 72 hours before explantation, or wild-type tumor cells were used. IR of the formed tumor spheroid was performed on developmental day 12. The experiment was terminated on developmental day 14. Tumor growth and number of vessels were assessed by imaging on developmental days 11 and 14. The formed tumor spheroid was cut out and fixed with 4% para-formalin (Merck; 30525–89–4) including the adjacent CAM and prepared for histology. For treatment of tumor spheroids with the ADAM17-inhibitory antibody MEDI3622 or the control antibody IgG1, 2 μL of 25 μmol/L PBS–antibody solution was injected in a major vessel on the spheroids present on the opposite site of the embryo. Tumor spheroids were sham-irradiated or irradiated with a single dose of 5 Gy using an image-guided small-animal radiotherapy platform (Precision X-Ray, X-Rad SmART) 225 kV unit with a dose rate of 3 Gy/minute, equipped with a cone beam CT (CBCT) scanner. Precise irradiation plans were designed with the corresponding SmART Plan software. Spheroids were localized in the planning CT with chicken bone fragments placed in defined distance from the spheroid. At least six biological replicates were performed for each treatment modality.

### Flow Cytometry

To determine cell surface levels of VEGF-A, cells were seeded at a density of 4 × 10^5^ cells per well in a 6-well plate and incubated at 37°C overnight. One hour after treatment with either IgG1 or MEDI3622 cells were (sham-)irradiated with 5 Gy. Twenty-four hours after irradiation, cells were harvested using citric saline (Merck; C3674) and blocked with 3% BSA/PBS (Biolabs; B9001S) for 15 minutes. After 15 minutes of staining with Zombie dye (1:200 in PBS, BioLegend; 423105), cells were incubated with a human anti–VEGF-A antibody (1:100 in PBS, Abcam, ab52917, RRID: AB_883427) for 1 hour, followed by incubation with goat anti-rabbit Alexa Fluor 594 (1:200 in PBS, Thermo Fisher; A-11012, RRID: AB_2534079). After each step, cells were washed twice in 1% BSA/PBS and centrifuged at 15,000 rpm at 4°C. From harvesting until measurement, cells were kept on ice. FACS (FACSCanto analyzer BD Biosciences) was used to measure the mean fluorescence intensity (MFI), and FlowJo software v5 (RRID:SCR_008520) was used for the analysis.

## Murine Tumor Models

To generate subcutaneous tumor xenografts, 4 × 10^6^ A549 cells were injected subcutaneously in the back of 8-week-old, female athymic CD1 nude mice (Charles River, IMSR CRL:086, RRID: IMSR_CRL:086) in a total volume of 150 μL. Tumor volumes were determined with a caliper according to the formula (*L* × *l*^2^)/2. Treatment started when tumors reached a volume of 300 mm^3^ ± 10%. To generate orthotopic xenografts, 4 × 10^6^ A549 cells transfected with SV40-luciferase vector (15) were injected via tail vein in 8-week-old, female athymic CD1 nude mice (Charles River) in a total volume of 150 μL. Tumor growth was followed by sequential bioluminescence measurements. Treatment started when the total photon emission (flux/s/cm^2^) reached 1 × 10^6^ flux/s/cm^2^ for the efficacy-oriented experiment (15–20 days following tumor cell injection) and 1.5 × 10^6^ photon flux/s/cm^2^ for the histologic endpoint 5 days after irradiation (20–25 days following tumor cell injection).

### *In Vivo* Treatment and Irradiation

Treatment of subcutaneous and orthotopic tumor xenografts consisted of injection of IgG1-control and ADAM17-inhibitory antibody injection (5 mg MEDI3622/kg bodyweight, i.p.), respectively, on two consecutive days, followed by (sham-)irradiation on day 3 and additional treatment with IgG1 control and MEDI3622 (5 mg/kg bodyweight, i.p.) on two consecutive days (days 4 and 5). Dosage of 5 mg/kg MEDI3622 was based on results and adjusted for the assessment of MEDI3622 in combination with irradiation (15). Five days after irradiation, tumors were harvested and fixed in 4% para-formalin (Merck; 30525-89-4) and prepared for histology. Tumors were sham-irradiated or irradiated with a single dose of 5 Gy using an image-guided small-animal radiotherapy platform (Precision X-Ray, X-Rad SmART) 225 kV unit with a dose rate of 3 Gy/minute, equipped with a CBCT scanner. Precise irradiation plans were designed with the corresponding SmART Plan software. The animals were kept under 3% isoflurane anesthesia in a feet-first prone position on the stage in the irradiator for imaging and treatment. Radiotherapy was applied with two opposing fields from top and bottom, sparing the spine of animals bearing subcutaneous tumors. Animals bearing orthotopic tumors only had the right thorax (sham-)irradiated with two opposing fields from top and bottom, spearing the heart and spine. For both models, a 8 × 12 mm collimator was used, with the long edge aligned to the animal stage. Animals were kept under 3% isoflurane anesthesia for imaging and treatment. All *in vivo* experiments were performed according to guidelines for the welfare and use of animals and approved by the Veterinäramt Kanton Zurich, Switzerland (ZH221/2019).

### *In Vivo* Bioluminescence Imaging and Analysis

A549 cells transfected with SV40-luciferase vector were used as described previously (16). Mice with orthotopic tumors derived from SV40-luciferase–expressing A549 cells were intraperitoneally injected with 150 mg/kg d-Luciferin (Perkin Elmer) 5 minutes prior to anesthesia. Sequential measurements of photon emission (photon flux/s/cm^2^) were acquired approximately 10 minutes after d-Luciferin injection with the IVIS200 (Xenogen) and analyzed with the Living Image Software (Perkin Elmer). The sequence with the highest photon flux was used for analysis. The values were normalized to the baseline value (*t* = 0).

### IHC

Immunohistologic endpoints were analyzed on formalin-fixed paraffin-embedded 4-μm tissue sections derived from tumor xenografts and tumor lesions in the lung derived from the orthotopic model and stained with hematoxylin and eosin (Abcam, ab245880) and anti-CD31–directed antibody (1:10, Dako; M0823, RRID: AB_2114471). Images were taken on a wide-field Nikon Eclipse TI microscope. Number of vessels and their diameter were counted and measured in ImageJ (RRID: SCR_003070) in at least 12 different fields in each xenograft.

### Transwell Migration Assay

For the transwell migration assay, 24-well transwell units (6.5 mm diameter) with 1 μm and 8 μm pore size PET membranes (Greiner Bio-One; 662610 and 662638) were used according to the manufacturer's instructions. Briefly, 3 × 10^5^ attracting cells (A549, A549_shNT, A549_shADAM17 or H358, H358_shNT, H358_shADAM17) were plated into a 24-well plate (bottom chamber) in 1,000 μL ECGM supplemented with 1% FBS and without growth factors. Simultaneously, 3 × 10^4^ endothelial cells (HUVECs or HPMECs) in 200 μL of ECGM supplemented with 1% FBS and without growth factors were seeded into transwell inserts (top chamber). The next day, medium was renewed (ECGM with 1% FBS, no growth factors) and the inserts and plates were sham-irradiated or irradiated with 5 Gy and inserts were immediately placed on the wells harboring the attracting cells. The coculture was maintained at 37°C in 5% CO_2_ for 24 hours. For quantification, cells from the top side of the insert were scrapped away with a cotton swap and inserts were then fixed in methanol/acetic acid (75%/25%, v/v), dried, and stained with DAPI (Invitrogen; D1306, 1:25,000) in 99% MeOH. Fluorescent microscopy pictures were taken (Leica 7000DT) and the migrated cells were counted manually in at least three images at 40× magnification per insert. For each experiment, at least three biological replicates were performed.

### Statistical Analysis

The data were analyzed with GraphPad Prism v7 (RRID: SCR_002798) and tested for normal distribution by using the Shapiro–Wilk normality test. For the direct comparison of two treatment groups, the two-tailed unpaired *t* test or Mann–Whitney test was applied. For the comparison of more than two treatment groups, one-way ANOVA test with Tukey posttest was performed. Error bars, SEM. *P* ≤ 0.05 was considered significant. *In vitro* data were collated from at least three biological replicates obtained from at least three independent experiments. For all experiments, *, *P* < 0.05; **, *P* < 0.01; ***, *P* < 0.001.

### Data Availability Statement

The data that support the findings of this study are available on request from the corresponding author (M. Pruschy).

## Results

### Irradiation Induces Endothelial Cell Migration in an ADAM17-dependent Manner

**FIGURE 1:**
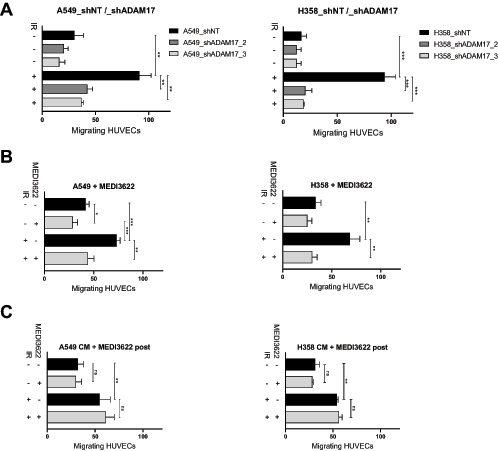
IR-induced endothelial cell (EC) migration is dependent on tumor cell located ADAM17 activity. Transwell migration assay of migrating endothelial cells in the top chamber and attracting irradiated NSCLC cells in the bottom attracting chamber. **A,** A doxycycline (Dox)-inducible shRNA system directed against ADAM17 (shADAM17_2 and shADAM17_3) or control shNT in the attracting A549 and NCI-H358 tumor cells was used to assess the effect of ADAM17 inhibition for attraction of migrating endothelial cells. **B,** The mAb MEDI3622 was used to downregulate ADAM17 on attracting tumor cells. **C,** To exclude a potential direct effect of MEDI3622 on the migratory capacity of the endothelial cells, MEDI3622 was only added to the CM after CM production by the attracting tumor cells. For all experiments, ECs were irradiated with 5 Gy prior to exposition to attracting tumor cells or CM thereof. The amount of migrated cells was determined after 24 hours. Bar graphs represent average number of migrated ECs counted in three randomly chosen fields/transwell of at least three independent biological replicates. *, *P* < 0.05; **, *P* < 0.01; ***, *P* < 0.001.

As part of an extended screening approach, we previously identified IR-dependent upregulation of ADAM17 sheddase activity and secretion of angiogenic factors from lung adenocarcinoma cells (10). To further investigate IR-dependent tumor to vasculature-oriented communication *in vitro*, a Boyden chamber transwell migration assay was developed with HUVECs migrating toward attracting lung adenocarcinoma tumor cells. Irradiation of A549 and NCI-H358 lung adenocarcinoma tumor cells enhanced migration of HUVECs in a dose-dependent way, indicating the release of soluble, promigratory factors from the irradiated tumor cells (Supplementary Fig. S1A and S1B). To determine a potential ADAM17 dependence of IR-induced migration, the transwell migration assay was performed with tumor cells, that were stably transfected with doxycycline-inducible ADAM17-directed shRNAs (shADAM17_2 and shADAM17_3; Supplementary Fig. S1C and S1D). Knockdown of ADAM17 in the attracting lung adenocarcinoma tumor cells A549 (A549_shADAM17_2 and _3) and NCI-H358 (H358_shADAM17_2 and _3) minimally reduced basal migration of HUVECs, but strongly abrogated migration of HUVECs toward irradiated ADAM17-knockdown lung adenocarcinoma cells in comparison with endothelial cells migrating toward ADAM17-proficient tumor cells (A549_shNT and H358_shNT; Fig. 1A). Of note and due to the minimal basal migratory capacity of nonirradiated HUVECs, the migration capacity was always assessed with preirradiated HUVECs (5 Gy; Supplementary Fig. S1E, see Materials and Methods), thereby also mimicking the *in vivo* situation of a coirradiated tumor vasculature. To assess the role of the IR-induced sheddase activity of tumor cell–expressed ADAM17 for HUVEC migration, the transwell migration assay was performed with nonirradiated and irradiated A549 and NCI-H358 cells, that were preincubated with the ADAM17-directed inhibitory antibody MEDI3622 (Supplementary Fig. S1F). Migration of HUVECs toward nonirradiated, but MEDI3622-preincubated, lung adenocarcinoma cells was only minimally affected. In contrast, inhibition of ADAM17 activity of irradiated tumor cells strongly abrogated IR-induced migration of HUVECs toward these MEDI3622-preincubated tumor cells (Fig. 1B). To exclude a direct antimigratory effect of MEDI3622 on HUVEC-expressed ADAM17, the transwell migration assay was also performed with conditioned media (CM), to which MEDI3622 was only added following CM collection (CM + MEDI3622 post). Migration of HUVECs exposed to CM, that was derived from irradiated tumor cells (A549, NCI-H358) was enhanced in comparison with HUVECs exposed to CM, derived from nonirradiated tumor cells. MEDI3622 did not abrogate the IR-induced capacity of the CM to increase HUVEC migration when added after CM collection (Fig. 1C). Corresponding experiments were also performed with HPMECs. Similar to HUVECs, migration of HPMECs was also increased in response to IR, but only toward irradiated ADAM17-proficient tumor cells (Supplementary Fig. S1G and S1H).

Overall, these complementary experiments demonstrate irradiation-induced shedding of ADAM17 substrates from tumor cells, which increase migration of endothelial cells *in trans*.

### Tumor VEGF is Expressed and Processed by ADAM17 in Response to Irradiation

**FIGURE 2:**
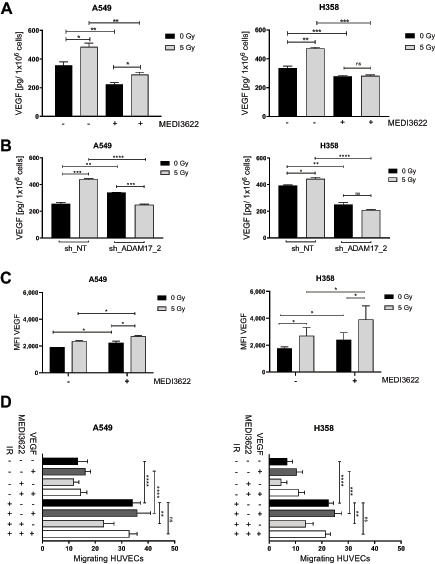
VEGF secretion is increased after irradiation in an ADAM17-dependent manner. Irradiation-induced VEGF secretion was investigated in A549 and NCI-H358 cancer cells. Supernatants were collected and analyzed for VEGF secretion 24 hours after irradiation by ELISA. **A,** Cells were pretreated with MEDI3622 1 hour prior to IR. **B,** ADAM17 was downregulated with a doxycycline (Dox)-inducible shRNA system targeting ADAM17 (shADAM17_2) or control (shNT). Bar graphs represent VEGF concentration normalized to viable cell count ± SD. **C,** Tumor cell–associated surface VEGF levels were quantified via FACS. Cells were pretreated with MEDI3622 1 hour prior to IR, fixed 24 hours thereafter and stained for VEGF. MFI was analyzed with a FACS (BD FACSCanto analyzer). **D,** Transwell migration assay of endothelial cells migrating to attracting tumor cells treated with IR alone or in combination with MEDI3622 and additional recombinant VEGF supplement. *, *P* < 0.05; **, *P* < 0.01; ***, *P* < 0.001; ****, *P* < 0.0001.

To determine a putative role of the VEGF for IR-and ADAM17-dependent HUVEC migration, expression and secretion of VEGF was analyzed on irradiated and ADAM17-inhibited tumor cells and in supernatants thereof. The level of soluble VEGF was significantly increased in the supernatant of irradiated A549 and NCI-H358 tumor cells in comparison to the supernatant derived from nonirradiated tumor cells. Cellular pretreatment of tumor cells with MEDI3622 slightly reduced basal and abrogated IR-induced release of VEGF into the supernatant (Fig. 2A; Supplementary Fig. S2A). Likewise, irradiation-induced secretion of VEGF was also suppressed in tumor cells expressing the ADAM17-directed shRNA construct in comparison to tumor cells transduced with the control shRNA construct (Fig. 2B; Supplementary Fig. S2B). Of note, and as previously observed by others, expression of VEGF was increased at the transcriptional level upon irradiation, which was not affected by MEDI3622 (Supplementary Fig. S2C; ref. 17). To quantify the cell surface levels of VEGF in response to treatment, cell surface VEGF was analyzed with a human anti-VEGF-A–directed antibody. An increase of VEGF-linked MFI was determined by flow cytometry on the surface of tumor cells 24 hours after irradiation, which was further upregulated in MEDI3622-pretreated cells (Fig. 2C). These results suggest that irradiation of tumor cells drives enhanced expression of VEGF at the plasma membrane with subsequent ADAM17-mediated shedding into the secretome, which is thereby abrogated in MEDI3266-pretreated cells. To compensate for MEDI3622-inhibited VEGF shedding during the transwell migration assay, exogenous VEGF was added to the secretome, which could fully reactivate HUVEC migration toward irradiated tumor cells (Fig. 2D).

### MEDI3622 in Combination with Irradiation is Antiangiogenic and Inhibits Growth of Tumor Spheroids and Xenografts

**FIGURE 3:**
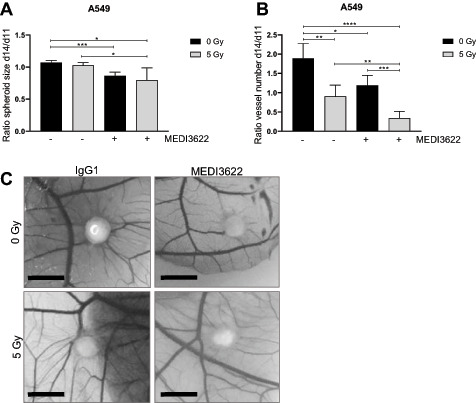
Tumor spheroid growth and vascularization on the CAM of fertilized chicken eggs is reduced by ADAM17 inhibition alone or in combination with irradiation. A549 lung adenocarcinoma cells were inoculated on the CAM in between major vessels on developmental day 9. Resulting spheroids were treated with MEDI3622 or control antibody IgG1 on day 11 and received (sham-) irradiation on developmental day 12. **A,** Spheroid size was measured on developmental day 11 (d11) and 14 (d14). Change in tumor spheroid size on d14 was related to size on d11. **B,** Spheroid-associated vessels were counted manually on d11 and d14. Change in spheroid-associated vessel number on d14 was related to spheroid-associated vessel number on d11. **C,** Representative images of tumor spheroids on d14. Scale bar, 15 mm. *, *P* < 0.05; **, *P* < 0.01; ***, *P* < 0.001; ****, *P* < 0.0001.

**FIGURE 4:**
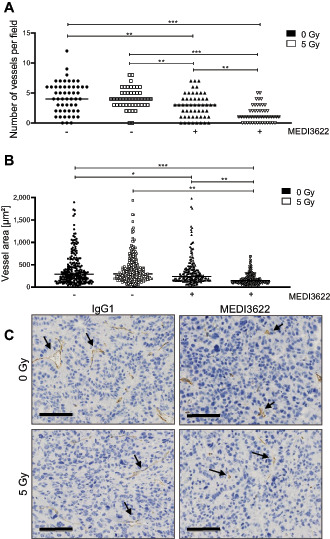
ADAM17 inhibition in combination with single dose of irradiation reduces vessel number and vessel size in a tumor xenograft model. **A,** Number of vessels within the tumor were counted manually in 12 randomly chosen fields/individual. One value indicates the average vessel number of one analyzed field **B,** Vessel area (μm^2^) was measured for each counted vessel (from **A**). **C,** Representative images of anti-CD31—stained formalin fixed tumor sections. Scale bar, 100 μm. *, *P* < 0.05; **, *P* < 0.01; ***, *P* < 0.001.

As previously investigated IR-regulated ADAM17 sheddase activity codetermines the intrinsic radiosensitivity of tumor cells. These endothelial migration–oriented results indicate an additional role of tumor cell–located ADAM17 toward the formation of a tumor vasculature. To initiate investigating the influence of ADAM17 activity on vessel formation *in vivo*, the CAM assay was established, which is often used to determine tumor angiogenic treatment responses (18). Doxycycline-preincubated shNT- and shADAM17- expressing A549 and H358 tumor cells were spotted onto the CAM of fertilized chicken eggs (developmental day 9) to form tumor spheroids, but interestingly only tumor cells proficient in ADAM17 activity were competent to form spheroids (>2,000 μm in diameter; Supplementary Fig. S3A). Thus, only spheroids derived from wild-type tumor cells (A549, H358) were treated in the later experiments with the control antibody IgG1 and the ADAM17-inhibitory antibody MEDI3622 (25 μmol/L, day 11), respectively, and irradiated (5 Gy) 24 hours thereafter. A significant tumor spheroid growth delay was induced on ADAM17 inhibition, which was further increased when combined with irradiation (Fig. 3A; Supplementary Fig. S3SB for NCI-H358). Vessel formation toward the tumor spheroids almost doubled over 72 hours (d11 to d14) in IgG1-treated tumor spheroids was halted in response to irradiation and only slightly increased on ADAM17 inhibition within the treatment period. Interestingly, the amount of vessels growing toward the spheroids was decreased in response to the combined treatment modality indicating a strong antiangiogenic effect of ADAM17 inhibition when combined with irradiation (Fig. 3B, ratio vessel number d14/d11; Supplementary Fig. S3SC for NCI- H358). Combined treated tumor spheroids also had a pale, almost translucent appearance in comparison to IgG1-treated control spheroids (Fig. 3C). Due to technical reasons, the microvessel density (MVD) could not be determined within the tumor spheroids growing on the CAM. Therefore, A549-derived tumor xenografts were analyzed from mice, treated with IgG1 control and MEDI3622 (5 mg MEDI3622/kg bodyweight, i.p., on two consecutive days), respectively, followed by locoregional (sham-)irradiation (day 3) and additional treatment with IgG1 control and MEDI3622 (5 mg/kg bodyweight, i.p.) on two consecutive days (days 4 and 5). Treatment was initiated when tumors reached a volume of 300 mm^3^ ± 10%. Seven days after treatment start, tumors were harvested for histologic analysis. Similar to the CAM-based results, each treatment modality alone reduced the MVD in comparison to IgG1 control–treated tumor xenografts. Furthermore, the combined treatment modality significantly pronounced this antiangiogenic effect (Fig. 4A). Treatment with MEDI3622 not only decreased the MVD but also the individual vessel size. Vessels in MEDI3622-treated tumor xenografts and even more so in tumors treated in combination with irradiation, were significantly smaller in comparison to vessels in untreated and irradiated tumor xenografts (Fig. 4B). Representative images of tumor cross sections stained with anti-CD31 are depicted in Fig. 4C. Tumor sections were also stained for the expression status of CAIX, which is often used as an endogenous marker for tumor hypoxia. In comparison to tumor sections derived from untreated mice, a modest and intermediate increase in CAIX expression could be determined in tumors derived from mice treated with MEDI3622 and irradiation, respectively, alone. The CAIX expression status was highest in tumors treated with MEDI3622 in combination with irradiation, which correlates with the most strongly reduced MVD and vessel areas observed on combined treatment (Supplementary Fig. S4A). Of note, the combined treatment modality already induced a significant tumor growth delay during this short treatment and observation period until tumor harvesting in comparison to untreated tumors and tumors that only received one treatment modality alone (Supplementary Fig. S4B). On the other hand, pretreatment of tumor cells with MEDI3622 *in vitro* only minimally sensitized A549 and NCI-H358 tumor cells to irradiation (Supplementary Fig. S4C).

### Irradiation in Combination with ADAM17 Inhibition Controls Tumor Growth in an Orthotopic Lung Tumor Model

**FIGURE 5:**
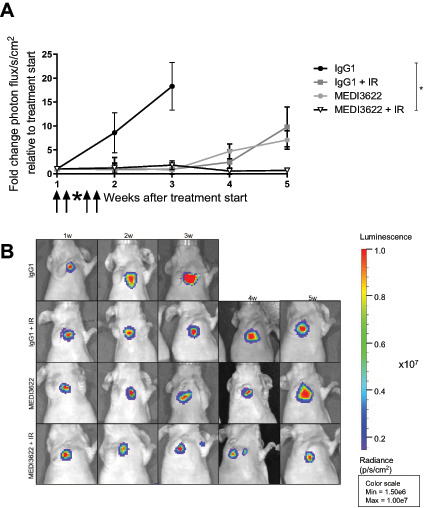
ADAM17 inhibition in combination with single-dose IR reduces tumor growth in an orthotopic lung cancer model. Lung cancer lesions were generated by injecting luc-expressing A549 lung cancer cells via tail vein. Tumor growth was monitored via IVIS by sequential measurement of bioluminescent signal. **A,** Fold change in photon flux/s/cm^2^ after treatment start. **B,** Representative bioluminescent images of mice harboring luc-expressing A549 lung cancer lesions. *, *P* < 0.05.

**FIGURE 6:**
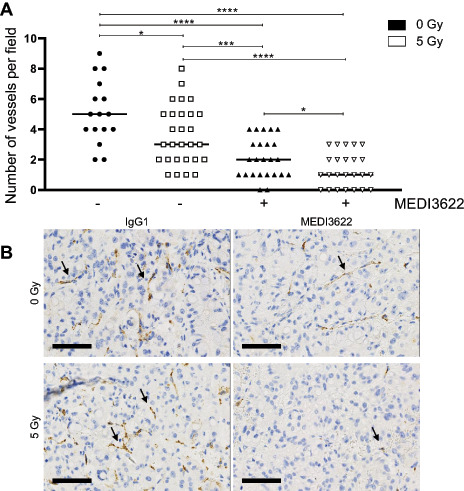
Tumor vessel number decreases after IR in combination with ADAM17 inhibition in an orthotopic lung cancer model. **A,** Number of vessels within the tumor lesion were counted manually in five randomly chosen fields/individual. One value indicates the average vessel number of one analyzed field. **B,** Representative images of anti-CD31–stained formalin-fixed tumor sections. Scale bar, 70 μm. *, *P* < 0.05; ***, *P* < 0.001; ****, *P* < 0.0001.

To determine the efficacy of the novel ADAM17-directed inhibitory antibody MEDI3622 in combination with IR in a more advanced tumor model, stably transduced firefly luciferase–expressing lung adenocarcinoma cells (A549-Luc) were tail-vein injected into mice to form orthotopic lung tumor nodules. Mice were treated with the same regimen as used for the subcutaneous tumor model. Irradiation was applied with two opposing fields of 8 × 12 mm to the right half of the thorax using a small-animal image-guided radiotherapy platform and the corresponding software SmartPlan to ensure precise dose deposition. Treatment started when thorax-derived bioluminescence reached a predefined signal of 1 × 10^6^ photon flux/s/cm^2^ and tumor growth was followed by weekly bioluminescence measurements. IgG1 control–treated animals reached survival endpoints three weeks after treatment start whereas irradiated and animals treated with MEDI3622 alone demonstrated tumor growth control over approximately three weeks with tumor recurrence thereafter. Extended tumor growth control over the entire observation period (five weeks) was induced in mice treated with MEDI3622 in combination with irradiation (Fig. 5A), demonstrating the potency of this combined treatment modality. Representative images of mice 5 weeks after treatment start (3 weeks for IgG1 control group) are depicted in Fig. 5B. Of note, analysis of tissue morphology adjacent to the tumor did not reveal any normal tissue toxicities in response to the different treatment modalities, and determination of body weight did not show any weight loss with MEDI3622 during the course of the treatment and the follow-up period in the two tumor models (Supplementary Fig. S4D). Orthotopic tumors were also harvested on day 7 following treatment start and histologic sections were stained with an anti-CD31–directed antibody. In comparison to IgG1 control–treated tumors, the MVD was reduced in tumors treated with MEDI3622 and IR, respectively, alone. Furthermore, the combined treatment modality also significantly pronounced this antiangiogenic effect in this orthotopic tumor model (Fig. 6A and B).

## Discussion

We previously provided novel insights into IR-mediated activation of ADAM17 with subsequent substrate shedding in response to irradiation, and demonstrated that inhibition of ADAM17 sensitizes otherwise treatment-resistant lung carcinoma cells to IR. This autocrine effect is partially mediated via downregulated ligand shedding of the ErbB family members such as TNFα, EGF, and TGFα, which decreases ErbB-receptor tyrosine kinase signaling and subsequently enhances sensitivity to IR-induced DNA damage (10, 19–22). As part of this initial secretome search, factors were also identified that suggested IR-mediated paracrine communication (10). Using the novel ADAM17-directed inhibitory antibody MEDI3622, we have now demonstrated a strong antiangiogenic effect in response to the combined treatment modality of radiotherapy with MEDI3622 in a subcutaneous and an orthotopic lung carcinoma model, which was corroborated with the angiogenesis-oriented CAM assay. The antiangiogenic treatment response also strongly correlated with efficacy-oriented endpoints, which were most prominent in the orthotopic tumor model.

Several small molecular compounds were previously identified as ADAM17-specific inhibitors, which also induced an increased tumor response when combined with chemo- and radiotherapy. However, these inhibitors also demonstrated an increased affinity toward the related matrix metalloproteinase ADAM10. Only in the last decade, highly specific anti-human ADAM17-directed antibodies were developed binding ADAM17 in subnanomolar ranges (23). Characteristic for MEDI3622 is its high target sensitivity as it recognizes the surface loop sIVa- sIVb β-hairpin on the M-domain, unique for ADAM17 (14). MEDI3622 was previously investigated alone and in combination with anti-EGFR–targeting agents in preclinical colorectal and esophageal tumor models demonstrating promising antitumor activity (15). Of interest, MEDI3622 also disrupts ADAM17-mediated immunomodulatory processes. Inhibition of ADAM17-mediated CD16A shedding on NK cells by MEDI3622 was thereby shown to augment NK-cell stimulation via tumor targeting mAbs (24). Furthermore, tumor-unrelated inflammatory processes are also reduced on treatment with MEDI3622 (25, 26).

Our new efficacy-oriented results in the subcutaneous and orthotopic lung tumor models corroborate our former *in vivo* data, which were performed with the small molecular compound TMI-001 and demonstrate, to the best of our knowledge for the first time, that ADAM17 inhibition with the novel ADAM17-inhibitory antibody MEDI3622 represents a promising approach for a combined treatment modality with radiotherapy. Paracrine communication in-between tumor and endothelial cells was mechanistically investigated using a complementary *in vitro* dual chamber approach. Irradiation-induced migration of endothelial cells was strongly dependent on tumor cell–derived VEGF. Furthermore, IR-induced and VEGF-mediated intercellular communication required release of VEGF in an ADAM17-dependent manner and could be abrogated through inhibition of ADAM17 in the attracting tumor cells. IR-induced expression and secretion of VEGF has been well investigated by our own and previous studies by others (see below). Our new results now indicate a multistep process in which irradiation initially increases expression and localization of VEGF at the plasma membrane as observed on the individual tumor cell level on ADAM17 inhibition. Because irradiation also increases ADAM17 activity, the amount of VEGF released from the plasma membrane into the supernatant is further elevated, thereby enhancing endothelial cell migration. VEGF complementation to supernatants of ADAM17-inhibited, attracting tumor cells strongly indicate that VEGF is the major driving force for IR-induced endothelial migration. However, we cannot exclude additional factors required for this intercellular communication. Unfortunately, VEGF was below the technical detection limit in the blood of tumor-bearing mice and thus the *in vivo* antiangiogenic effect by MEDI3622 could not be directly attributed to VEGF dysregulation in response to treatment.

While enhanced expression and secretion of VEGF in response to external stress, including irradiation, has been well investigated on the quantitative level, surprisingly less is known about VEGF release on the mechanistic level. Secreted VEGF is proteolytically released from the extracellular matrix and heparan sulfate proteoglycans via cleavage of its C-terminal heparin binding domain. Thereby, a gradient of soluble VEGF is formed, which can bind to its cognate tyrosine kinase receptor on endothelial cells. However, inhibition of ADAM17 might also indirectly lead to a decrease in VEGF secretion and release in response to irradiation. ADAM17-mediated signaling might affect IR-induced VEGF-transcription, intracellular transport, plasma membrane localization, release, and putative endocytosis. Our FACS-based quantification of tumor cell associated surface VEGF levels in response to irradiation alone and in combination with MEDI3622 thereby do not mechanistically dissect these processes but support and complement our new data on ADAM17-dependent irradiation-induced release of VEGF (Fig. 2A–C). Detailed investigations are now required to determine whether VEGF is a direct substrate of ADAM17, whether additional proteolytic intermediates are involved in the ADAM17-dependent release of this major proangiogenic growth factor or at which signaling level ADAM17 regulates IR-enhanced VEGF release. More important, our results indicate that MEDI3622 reduces primary IR-induced proangiogenic VEGF release from tumor cells and thereby abrogates its proangiogenic and tumor vasculature–protective effect. The relevance of tumor-derived VEGF is also demonstrated as part of our *in vitro* experiments with shRNA-downregulated ADAM17 activity and with the CAM assay.

Interestingly, initial IR- and ADAM17-dependent proangiogenic VEGF release might be further propagated on the endothelial cell level in an ADAM17-dependent manner. VEGF was demonstrated to increase ADAM17 activity in endothelial cells via VEGFR/ERK/MAPK pathway activation with subsequent shedding of endothelial cell–located stimulatory factors (27). Furthermore, endothelial ADAM17-activity also reduces expression of the naturally occurring inhibitor of angiogenesis thrombospondin 1 (28).

Combined treatment modalities of radiotherapy with antiangiogenic agents represent a promising treatment approach, which was previously investigated with different classes of compounds and which is based on multiple in part opposing rationales. Inhibitors of angiogenesis induce endothelial cell apoptosis, might enhance the fragility of the tumor vasculature and thereby increase the efficacy of radiotherapy despite a slight rise in tumor hypoxia. Furthermore, inhibitors of angiogenesis might also normalize the tumor vasculature, create a window of improved tumor oxygenation, and thereby sensitize to radiotherapy (29–36). VEGF was widely accepted to be a critical regulator of angiogenesis and a promising target for tumor vasculature normalization (37). For example, the humanized anti-VEGF-A antibody Avastin was tested in combination with conventional chemotherapies or radiotherapy for several tumor entities and it became more evident that the success of antiangiogenic treatment with VEGF blockade was highly dependent on the tumor type. Combination of Avastin with chemotherapy in gastric cancer significantly improved progression-free survival and overall response and similar results were demonstrated in breast cancer metastases combined with paclitaxel, in NSCLC, in combination with cisplatin/gemcitabine and with radiotherapy (38–42). However, despite the successes of antiangiogenic treatment via VEGF blockade, side effects as hemorrhage, venous thromboembolism, vascular regression with subsequent increase in metastasis incidence, adaptive resistance and limiting normal tissue toxicities on the clinical level, ask for new treatment combinations exploiting the rationale for combining radiotherapy with antiangiogenic agents (43–47). Of note, inhibition of ADAM17 primarily reduced IR-enhanced VEGF release and, thus, MEDI3622 does not target VEGF on the systemic level. As such, a differential level of normal tissue toxicities than with Avastin is expected.

Surprisingly, MEDI3622 did not result in significant radiosensitization *in vitro* compared with a previously demonstrated increase in radiosensitivity on the unicellular level by ADAM17 knockdown using ADAM17-targeting siRNA (10). This differential response could be due to the differential protein quantity of ADAM17 localized at the plasma membrane in response to the two different targeting approaches. The cytoplasmic domain of ADAM17 interacts with multiple other proteins, which also coregulate extracellular ADAM17 metalloproteinase activity, but a differential protein level might also affect the cytoplasmic milieu on its own. Furthermore, reports also demonstrate cytoplasmic localization of ADAM17 (not targetable by MEDI3622) in different tumor entities, but its cytoplasmic role has not been further investigated (48–50). Radiosensitization by the cell-permeable small-molecule ADAM17 inhibitor TMI-005 on the unicellular level also suggests an additional role of ADAM17, which cannot be targeted by inhibition of its metalloproteinase activity once it is located at the extracellular site. Radiosensitization by MEDI3622 on the unicellular level has not been investigated in great detail. As such, it cannot be excluded that the ideal pharmacokinetic/pharmacodynamic conditions to sensitize for irradiation on the unicellular level have not been identified yet. More importantly and besides the differential radiosensitizing effects on the unicellular level, both targeting approaches (ADAM17-directed inhibition with MEDI3622 and downregulation using an shRNA approach) reduce the (IR-induced) release of VEGF and subsequent HUVEC migration.

Overall, we here successfully demonstrate the efficacy of the combined treatment modality of radiotherapy with the novel ADAM17-directed inhibitory antibody MEDI3622 for lung adenocarcinoma. Previous investigations suggested ADAM17 as a promising target for radiosensitization on the unicellular level. Our mechanistic-oriented results suggest an additional strong antiangiogenic response of relevance for this combined treatment modality to be investigated also for other tumor entities.

## Supplementary Material

Supplementary Data Supplementary figures plus legends
