# Supplementary material for: The ADAM17-directed Inhibitory Antibody MEDI3622 Antagonizes Radiotherapy-induced VEGF Release and Sensitizes Non–Small Cell Lung Cancer for Radiotherapy
Source: Cancer Res Commun. 2021 Dec 22;1(3):164–77. doi: 10.1158/2767-9764.CRC-21-0067 (PMC9973400; doi:10.1158/2767-9764.CRC-21-0067)
Supplement: Supplementary Data — Supplementary figures plus legends [file crc-21-0067-s01.pdf]

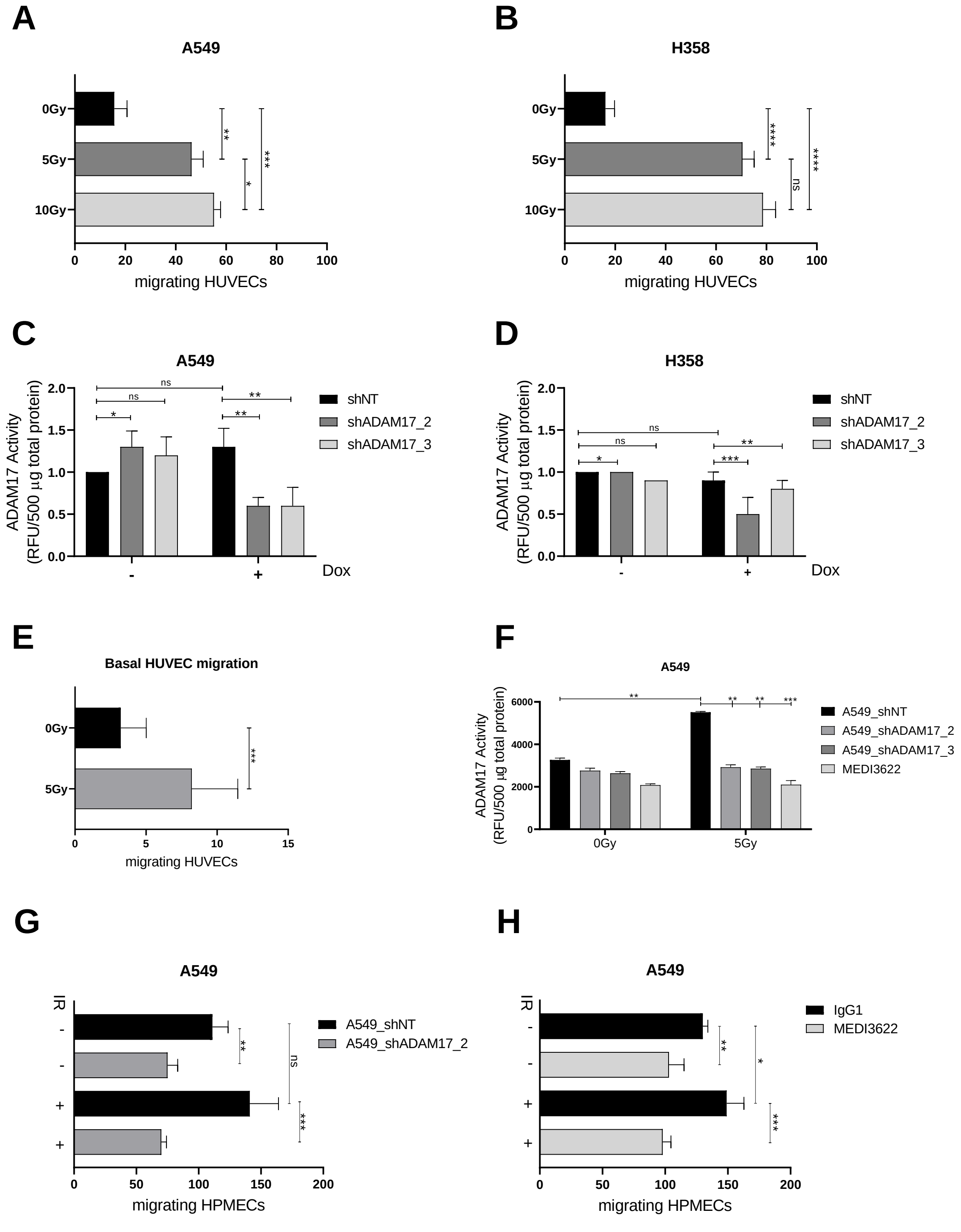

Supplementary Figure S1

A, B, Transwell migration assay of endothelial cells (ECs) migrating towards attracting A549 and NCI-H358 adenocarcinoma cells treated with increasing doses of irradiation (0, 5 and 10Gy). **C, D,** Doxocycline-inducible shRNA system targeting ADAM17 (shAdAM17_2, shADAM17_3) efficiently downregulates ADAM17 activity compared to control (shNT) in A549 and NCI-H358 adenocarcinoma cells**. E,** Basal migration of human umbilical vein endothelial cells (HUVECs) after irradiation with 0 and 5Gy. **F,** ADAM17-activity in A549 tumor cells after irradiation with 0 and 5Gy pretreated with MEDI3622 1h prior to IR and in cells expressing the control (shNT) or ADAM17-targeting construct (shADAM17_2 and shADAM17_3). **G,** Transwell migration assay of human pulmonary microvascular endothelial cells (HPMECs) towards attracting A549 tumor cells expressing the control (shNT) or ADAM17-targeting construct (shADAM17_2) or **H,** pretreated with MEDI3622 1h prior to irradiation.

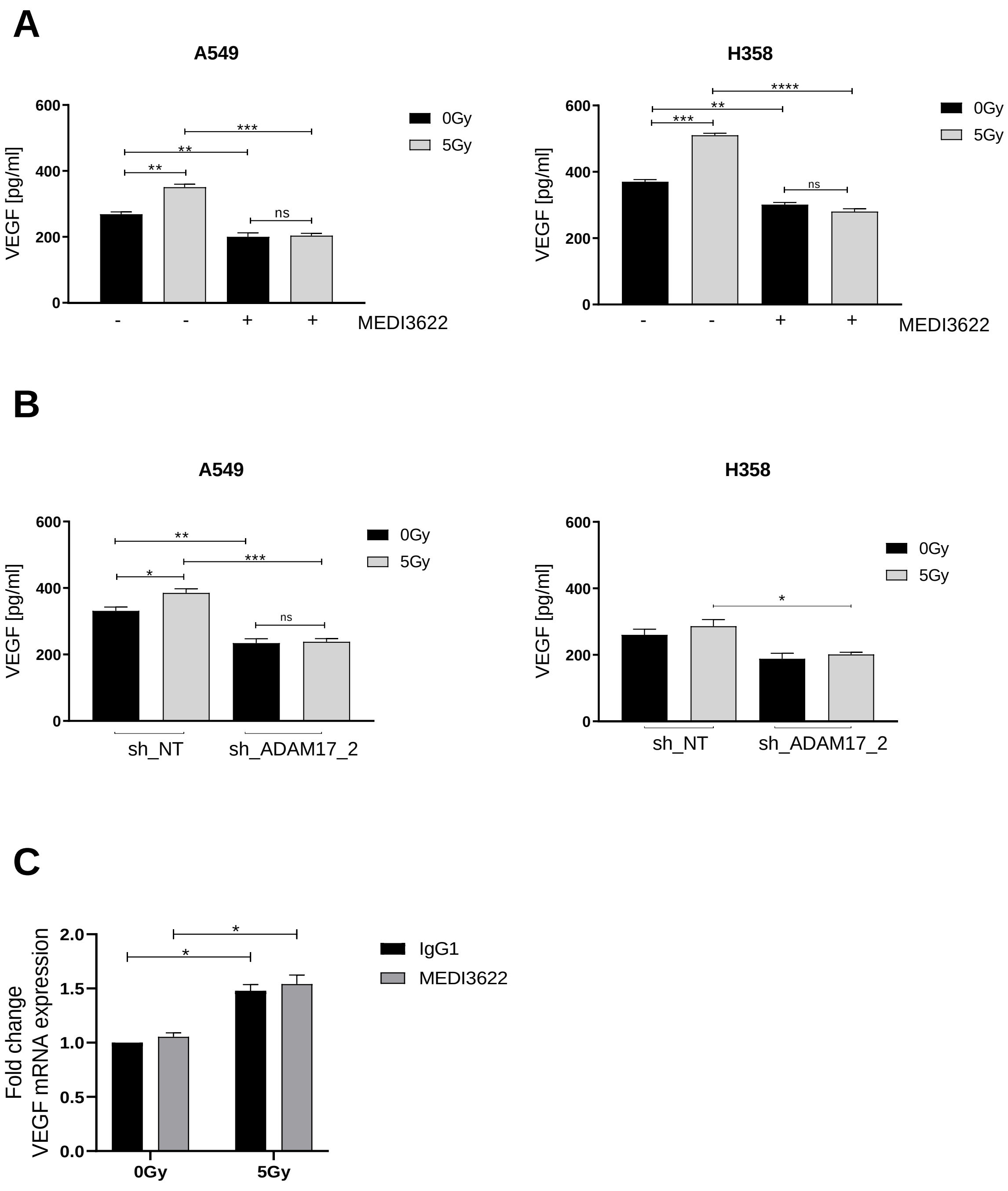

Supplementary Figure S2.

IR-induced VEGF secretion was investigated in A549 and NCI-H358 tumor cells. Supernatants were collected and analyzed by ELISA for VEGF secretion 24h after irradiation. ADAM17 activity was downregulated either using a doxocycline-inducible shRNA system targeting ADAM17 or the monoclonal antibody MEDI3622. **A,** absolute values of soluble VEGF in the supernatant of tumor cells pretreated with MEDI3622 and **B,** absolute values of soluble VEGF in the supernatant of A549 and NCI-H358 tumor cells expressing the ADAM17-directed shRNA construct. **C,** VEGF mRNA expression in A549 tumor cells 24h after irradiation alone or MEDI3622 treatment 1h prior to IR.

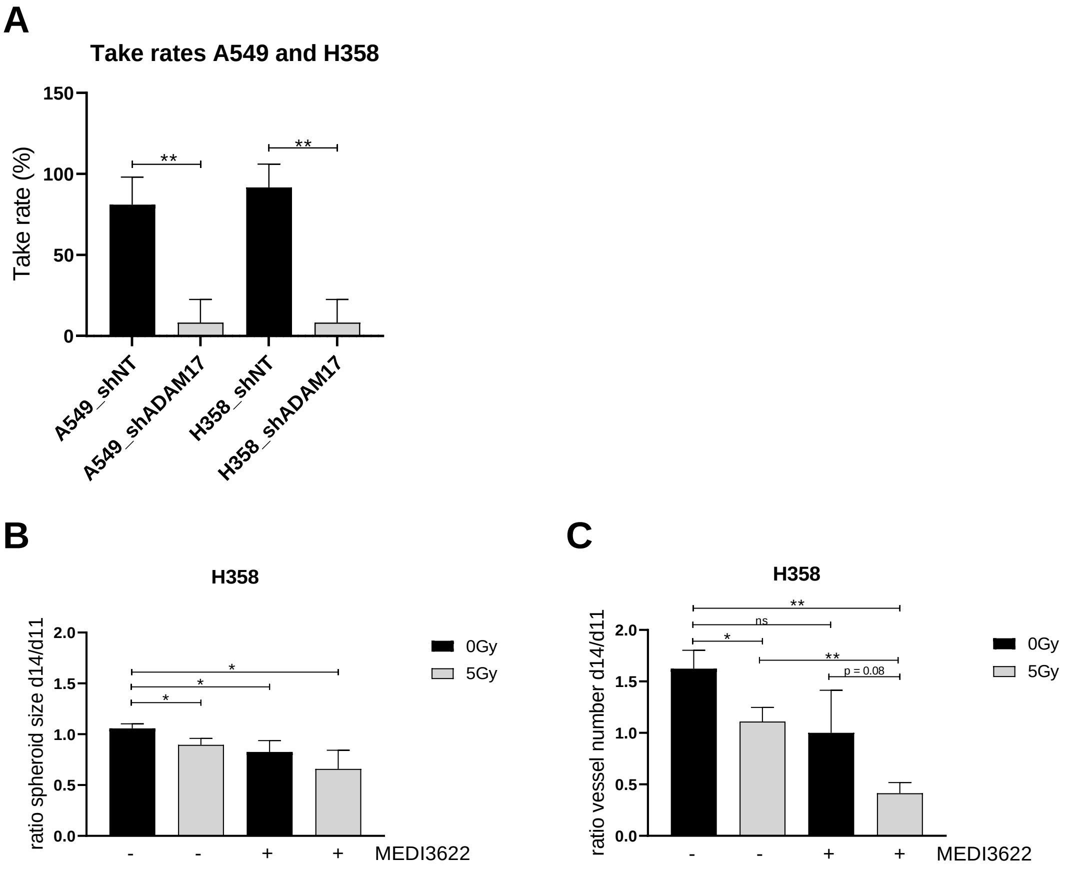

Supplementary Figure S3.

A, A549 and NCI-H358 adenocarcinoma cells expressing the doxocycline-induced control (shNT) or ADAM17-directed (shADAM17) shRNA construct were inoculated on the CAM in between major vessels on developmental day 9. Spheroid formation (% take rate) was recorded on developmental day 14. Cells were doxocycline-induced 72h prior to inoculation. **B, C,** NCI- H358 tumor cells were inoculated on the CAM in between major vessels on developmental day 9. Resulting spheroids were treated with MEDI3622 or control antibody IgG1 on day 11 and received (sham-) irradiation on developmental day 12. Spheroid size was measured, and spheroid-associated vessels were counted on developmental day 11 (d11) and 14 (d14). Change in tumor spheroid size and tumor spheroid-associated vessels on d14 was related to size and vessel number on d11.

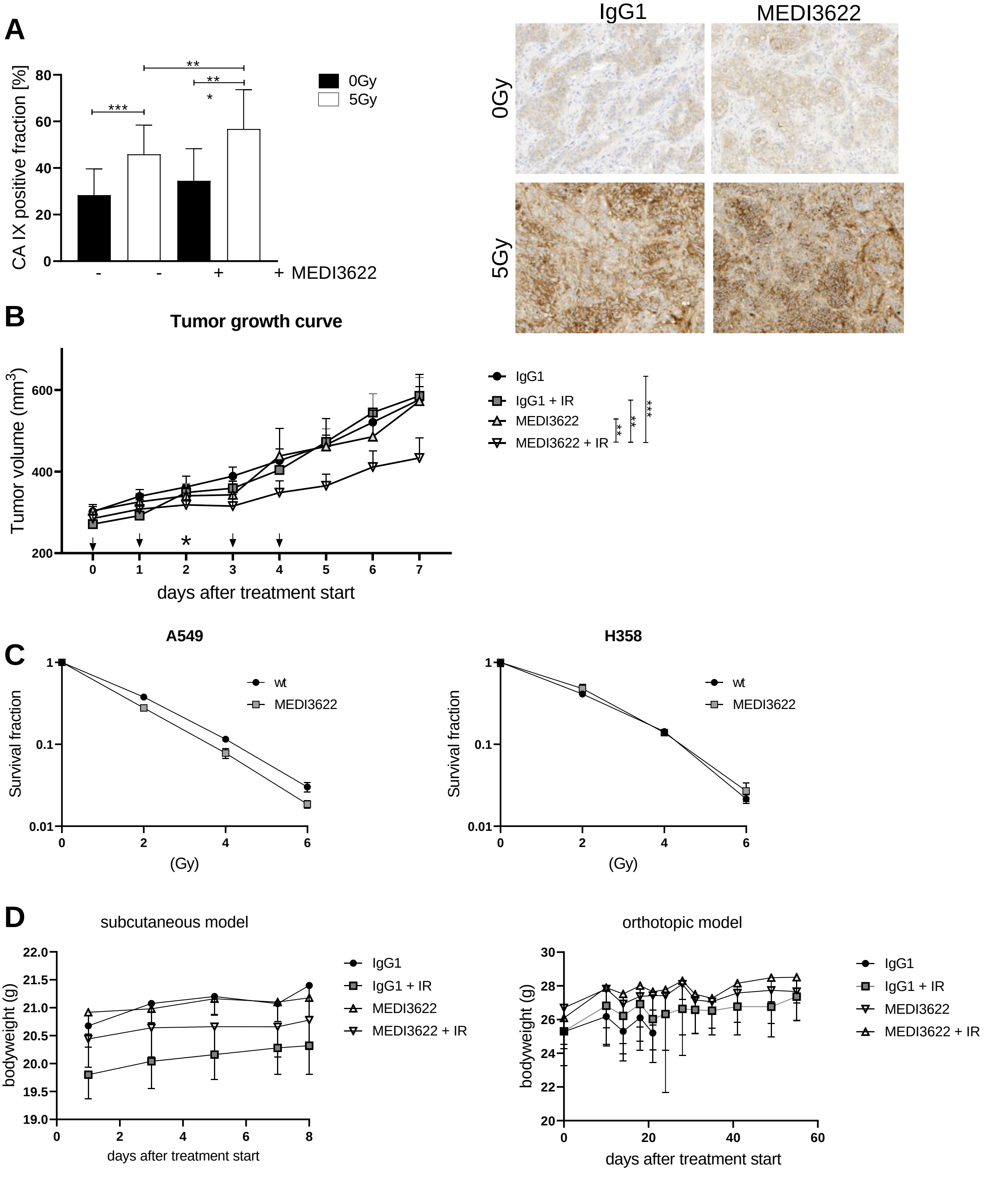

Supplementary Figure S4.

Treatment started when tumor xenografts reached 300mm^3 ± 10% and consisted of injection of IgG1-control and ADAM17-inhibitory antibody injection (i.p. 5mg MEDI3622/kg bodyweight), respectively on two consecutive days, followed by (sham-) irradiation on day 3 and additional treatment with IgG1-control and MEDI3622 (5mg/kg bodyweight) on two consecutive days (day 4 and 5). 5 days after irradiation, tumors were harvested and fixed in 4% para-formalin, prepared for histology, and stained against the vasculature marker CD31 and the hypoxia marker CAIX. **A,** CAIX positive fraction was determined using QuPath in 9 randomly chosen fields / individual. **B,** Tumor growth curves over the time course of the treatment until day of harvest. **C,** Clonogenic cell survival of A549 and NCI-H358 tumor cells after treatment with increasing doses of IR in combination with MEDI3622 1h prior to IR. **D,** Mouse bodyweight development during the treatment course and follow-up period of the subcutaneous and the orthotopic lung cancer model.
